# Effect of maturity timing on the physical performance of male Polish basketball players aged 13 to 15 years

**DOI:** 10.1038/s41598-021-01401-4

**Published:** 2021-11-10

**Authors:** Karol Gryko

**Affiliations:** Department of Sport Games, Józef Piłsudski University of Physical Education in Warsaw, Marymoncka 34, 00-968 Warsaw, Poland

**Keywords:** Computational biology and bioinformatics, Physiology, Health care

## Abstract

The aims of this study were (i) to identify the motor potential and basic anthropometric characteristics of Polish basketball players aged 13 to 15 years, (ii) to demonstrate the effect of maturity timing on the results achieved in motor tests and basic body composition parameters, and (iii) to determine which index contributes most to the prediction of performance in the individual tests of speed, jumping ability, agility, and endurance. The sample included 818 male Polish players. Analysis of values related to age-adjusted characteristics showed that in the under 13-year-old group, early maturers had significantly better results (except for stage 1 in the agility test) than average maturers. However, in the endurance test in the under 14- and 15-year-old groups (both distance covered and VO_2max_), the average maturers obtained higher values. Furthermore, maturity differentiation in the under 14- and 15-year-old groups significantly affected body size, 20-m sprinting time (under 14-year-old group only), and the results of all jumping tests. ANCOVA results (age, body height, and body mass as covariates) showed better results of early maturers in the under 13-year-old group. The opposite trend was observed in the under 14- to 15-year-old groups, where early maturing individuals performed worse in the running vertical jump (VJ) and endurance tests (both distances covered and VO_2max_). Maturity timing (VJ and VO_2_max), chronological age (5 m, 10 m, 20 m, agility, and VO_2max_ tests), body height (all tests), body mass (5 m), and the interaction between body mass and height (10 m, 20 m, agility, standing vertical jump, vertical jump) were significant (adjusted *R*^*2*^ = 0.08–0.25; *p* < 0.001) predictors of motor skills. These findings can be helpful in quantifying and controlling the results of youth sports programs adjusted to biological requirements used in the training process.

## Introduction

In basketball, success is determined by appropriate body dimensions^1^, technical^2^, motor^3–5^, tactical^6^, and psychological development^7^, and the physiological potential of players^8, 9^. Therefore, strength, power, agility, and speed are important attributes of basketball players^10^. Due to the demands of the sport, conducting physical fitness tests for basketball players should be based on movements similar to those occurring during the game and focus on multiple fitness elements simultaneously^11–13^. In the process of qualifying for the sport, coaches must look for young athletes with the potential to guarantee the achievement of maximum abilities in senior competitions^14^ to avoid early specialization and the unnecessary cost of training. Early success and specialization do not appear to predict late success in elite athletes across different sports^15^. In team sports (e.g. basketball), the period from 11 to 14 years is a period of intense development of the athlete, and talent should also be identified at this age^16^ and particularly consider biological maturity^17^.

Maturity status (early, on time, late, mature based on skeletal age, stage of puberty) refers to the state of biological maturation of an individual at the time of observation, whereas maturity timing refers to the ages when specific maturational events are attained (ages at peak height velocity and menarche)^17, 18^.

Biological maturation is significantly associated with adolescent growth and functional performance, resulting in significant performance differences between boys of the same chronological age (CA)^19^. The factors that influence performance at a young age may differ from those that influence the success of adult athletes^20^. The birth dates of athletes influence selection but do not determine the career development of professional athletes^21^. In contrast, biological maturation is a key factor for achieving higher levels of performance^21^. Therefore, when assessing the physical fitness of adolescents, an attempt should be made to determine the biological age^22^. The number of years from the age at peak height velocity (YAPHV) is a good indicator of the maturity of boys aged between 12 and 16 years and can be calculated using their CA and simple anthropometric measurements^23^.

Changes in body structure and neuromuscular and cardiorespiratory systems associated with the processes of growth and puberty of young basketball players strongly affect motor performance^17^. Strength and motor skills generally improve with age during middle childhood and adolescence, but the generally accepted pattern does not necessarily apply to all motor tasks. Running speed increases from 5 to 18 years of age in boys, whereas acceleration improves in adolescents after the age of 13 years^17^. Furthermore, the best period for the development of jumping ability in boys is the age of 13–15 years^16^, and the values achieved for vertical jumps (VJs) depend mainly on explosive strength^24^.

According to scientific reports, biological maturity is not only a factor that affects the physical performance of young basketball players^25, 26^ but is also a predictor of team performance^27^. Game performance results showed that post-pubertal players outscored others in blocks, but pubertal players were better in assists, assist-turnover ratio (Ast:TO), and steal-turnover ratio (Stl:TO)^28^. In addition, early maturers played significantly fewer minutes during European championships compared with national championships, whereas average maturers scored significantly more points and performed more assists during national championships^29^. In another study^25, 30^, the findings favored older basketball players in most biological variables (body height and mass, maturity, arm span, hand length, hand breadth, fat-free mass, and years of training experience), physical performance (mainly endurance, static muscular strength, vertical jumping, throwing, sprinting, and agility), and playing skills (shooting, passing, dribbling, capacity to move defensively, and slalom sprinting and dribbling). However, differences in physical fitness and technical skills (found only for slalom sprint test) between age groups were attenuated when adjusted for biological maturation and training experience^30^.

Furthermore, other studies have demonstrated that the results achieved in physical fitness tests were independent of differences in maturation, especially when differences in body size were taken into account^31^. Štrumbelj and Erčulj^32^ found that when evaluating young talent, the main predictive attributes were speed and agility (for predicting current abilities) as well as body height and growth potential (for predicting potential abilities), but expert assessment should also be considered. Some studies have shown that predicted maturity offset was negatively correlated with the 20 m sprint test^26^. Recent scientific reports conclude that somatic maturation is a strong predictor of variables derived from repeated power ability (RPA)^33^, especially concerning the lower body. Pubertal players exhibited greater aerobic fitness than late pubertal and post-pubertal players, but late pubertals outperformed their counterparts in terms of upper body power^28^.

Research that takes into account the biological maturity of individuals of the same chronological age avoids situations in which coaches focus on early maturing children due to their physical advantage over peers^27, 34–36^. Such players often have more opportunities to play during games at the expense of those who may develop their potential much later. This is a disadvantageous phenomenon for both early maturers (domination in the youth group may cause withdrawal from the sport at the moment when the physical potentials become even) and late maturers (who are discouraged due to the feeling of being weaker, worse, receiving less time for playing during the game) as it very often constitutes a serious barrier to the development of technical and tactical skills^17, 21^. As shown in various sports, the earlier sport selection occurs, the less accurate it is^34^. Therefore, in their work with young basketball players, coaches should consider the above observations^34, 37^ because it has been shown that monitoring player attributes is particularly important during periods of accelerated biological development to control training adaptations, reduce the risk of injury, and consequently increase the effectiveness of coaching^30, 38^.

Given the available research findings, there is a strong need for physical fitness testing of basketball players using large research samples, especially to identify talents^39, 40^. Previous basketball studies on the issues discussed in the present study have often been carried out for small samples or included athletes at a lower level of performance. Research based on a large sample size may be helpful in understanding the effects of maturity timing in a more precise way. Furthermore, it can also help interpret better the results achieved by young players during their training process, thus contributing to talent development^38^.

Assuming that a relationship exists between current maturity timing and the results achieved in motor tests and basic anthropometric characteristics, we conducted a study of a very large population of young basketball players aged 13–15 years training in Polish sports clubs. This is the first study of this type in the Polish context, which makes it unique. The first aim was to identify the motor potential and basic anthropometric characteristics. The second aim of the study was to demonstrate the effect of maturity timing on the results achieved in motor tests and basic body composition parameters. Finally, the third aim was to determine which index contributes most to the prediction of performance in the individual tests of speed, jumping ability, agility, and endurance.

## Methods

### Participants

The study examined 818 male basketball players aged 13 years (n = 233; age: 13.0 ± 0.3; basketball experience: 2.9 ± 0.9), 14 years (n = 364; age: 14.0 ± 0.3; basketball experience: 3.5 ± 1.2), and 15 years (n = 221; age: 14.7 ± 0.3; basketball experience: 4.2 ± 1.4). All examined athletes belonged to the Caucasian ethnic group. The players were members of 42 sports clubs competing in national championships in the under 13 and 15 years of age categories. The under 14 boys also participate in national championship games at the club and regional competition levels. This group includes basketball players who were part of the national team in their age category (under 14 years, n = 69; under 15 years, n = 63). At this training stage, all basketball players were characterized by a similar training volume, i.e., a total of 8 h 45 min per week (3 technical training sessions, 1.5 h each, 3 strength and conditioning sessions, 45 min each, and 2 h a week playing games). The players’ training experience and volume, were obtained from self-report questionnaires and cross-checked with registration histories, available from the subsystem of the Polish Basketball Association. The examinations were conducted between 2017 and 2020, during the same periods from November to February to complete the measurements before the play-off phase, which began in March.

### Procedure

All of the participants and legal guardians were informed in writing about the aims, benefits, and procedures of the research project, as well as the possibility to withdraw from the study at any moment without providing an explanation. The exclusion criteria included contraindications for the basic anthropometric measurements (inability to maintain the initial position of the body for measurements, balance disorders, lack of consent to participate in palpation measurements, and uncovering the body for measurements). Any injury or trauma also caused exclusion from the study. Twelve people were excluded or did not participate in the examinations due to lack of consent. The research was conducted in accordance with the approval from the local Ethics Committee for Scientific Research of the University of Physical Education in Warsaw (SKE 01-28/2016), and the study was completed according to the rules and regulations of the Declaration of Helsinki^41^.

### Biological maturation

The age at PHV (APHV) of the basketball players was estimated by subtracting the maturity offset from chronological age at the time of measurement^23^. The predicted maturity offset (YAPHV) was calculated as $-7.999994 +\left(0.0036124 \times [\mathrm{age}*\mathrm{stature}]\right)$, where the standard error of the equations was 0.542 years^42^. This equation was derived after calibrating the original equation provided by Mirwald et al.^23^. Early maturers, average maturers, and late maturers were defined as players with an estimated APHV of less than 13.1 years, 13.1–15.1 years, and greater than 15.1 years, respectively^22^. Given that no late-maturing cases were identified, early and average maturers were compared (Table 1).

**Table 1:** Distribution of stages of maturity status in the sample (n = 818) of male basketball players by age group.

Athletes	Maturity status	Sample size (n)	Frequency of occurrence (%)
Under 13	Average	120	51.5
	Early	113	48.5
Under 14	Average	178	48.9
	Early	186	51.1
Under 15	Average	130	58.8
	Early	91	41.2

### Measurements

Body height (cm) without shoes was measured with the head positioned to the Frankfurt plane, using a stadiometer (Seca 264, Seca GmbH & co. kg, Germany) with a precision of 0.1 cm, and standing reach measurements were performed (Seca 216, Seca GmbH & co. kg, Germany). Body mass was measured using a JAWON Medical X-Scan Plus II analyser (Certificate No. EC0197 for medical devices) with a 0.1-kg precision. The measurements were obtained using an anthropometry expert who holds an ISAK Level 1 accreditation according to the standards proposed by the International Society for the Advancement of Kinanthropometry (ISAK)^43^. All relative technical error of measurement (%TEM) values for intraobserver reliability of body height and weight were classified as acceptable (< 2%; range 0.19% to 0.87%; *R* ≥ 0.98)^43^.

After basic anthropometric measurements, standardized 20-min warm-up (slow jogging followed by static and dynamic stretching, and a series of submaximal sprints)^38^ was performed by strength and conditioning coach, and physical fitness tests were realized in the following order: speed, jumping ability, agility, and endurance. The fixed order of the individual tests allowed us to avoid performing two consecutive lower or upper body tests. Speed and power tests are performed first because they require maximum stimulation of the central nervous system^44, 45^. Each participant was verbally instructed and encouraged to give maximum effort. Then the participants were familiarized with the procedures by performing a trial (pretest). The athletes were allowed a 10-min passive rest between tests, as well as water breaks and extra rest time. Fitness level was measured in two stages, including the morning session (speed, jumping ability, agility) and the evening session (endurance), to ensure that adequate rest periods were maintained.

### Speed

Speed was analyzed based on the results of a 20-m sprint test and expressed as a split time at 5 m (starting speed) and 10 m in which players ran at full speed. Each athlete performed two trials, and the best trial was used as the test result^46^. Time (sec) was recorded using the photoelectric cells Fusion Smart Speed System (Fusion Sport, Coopers Plains, QLD, Australia). Compared to the dual-beam design, the system is equipped with single-beam gates to extend the battery life and simplify the setup. The Fusion Smart Speed System also uses an innovative error detection algorithm to reduce false triggers activating the gate. In the case of multiple triggers, the algorithm interprets the largest trigger as the actual event to activate the gate^47–49^. Photocells were installed at the starting line, at 5 m,10 m, and 20 m, and time measurements were performed with an accuracy of 0.001 s. Gates were set at a height of 1.0 m from the floor and separated by 1.5 m. The athletes started from a standing position with the preferred foot positioned in front. No bouncing or backward movements were allowed immediately before the sprint. The participants decided when to initiate the starting moment.

### Jumping

Both the standing vertical jump (SVJ) and a vertical jump (VJ) were measured using a yardstick vertical jump device^50–53^. The device measures the height to which players could push away small sticks placed horizontally on a pole during a jump. Reaching height was subtracted from the height reached while jumping. First, two standing countermovement jumps with arm swing were performed. Then, the player performed six attempts (with a run-up off two feet or one foot): two jumps with the dominant leg, two with the nondominant leg, and two with both legs with sufficient rest between jumps. The rest time between jumps was 20 s, and that between rounds was 5 min. The highest attempt was retained for analysis. This VJ protocol has established reliability^50–53^.

### Agility

**Figure 1:**
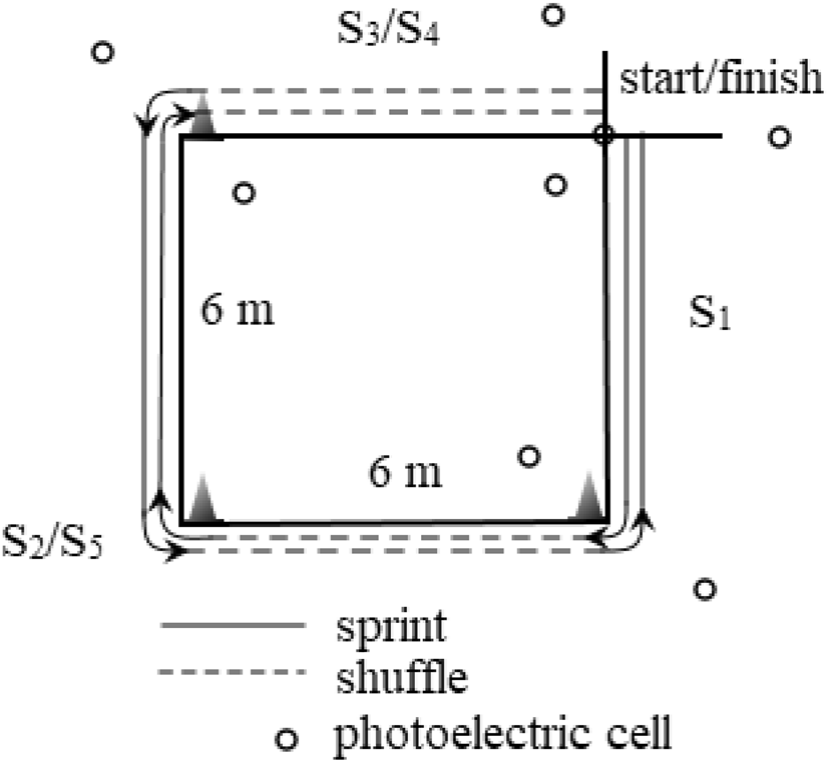
Agility test design. *S*_*1*_ stage 1 (front sprint); *S*_*2*_ stage 2 (shuffle and back sprint); *S*_*3*_*/S*_*4*_ stages 3 and 4 (shuffle); *S*_*5*_ stage 5 (front sprint and shuffle).

A diagram of the agility test is shown in Fig. 1. This is a modified Lane Agility Drill^3^ test, where only the length and width were changed to 6 × 6 m. Thus, the proportions between defensive shuffle and sprint are identical. A photocell at the change of direction line is placed at a distance of 1 m from this line. The test was repeated twice with a 10-min rest break in between to minimize fatigue. The best time of test completion was used for the result analyses.

### Endurance

The Yo-Yo Intermittent Recovery Test level 1 (Yo-Yo IR1) was used in an attempt to evaluate basketball players' endurance using a protocol reported in the literature^54, 55^. The total distance covered (m) during Yo-Yo IR1 was the main measure of performance and the maximum oxygen uptake (VO_2_max) was calculated according to the formula: VO_2_max = IR distance (m) × 0.0084 + 36.4^54, 56^.

### Statistical analysis

The normality of distribution was verified using the Shapiro–Wilk test, whereas the assumption of the equality of variance was tested using the Levene test. Interrater reliability agreement for all observations was assessed using Cronbach’s α statistic^57, 58^. A Cronbach’s statistic greater than 0.89 was obtained for all the datasets (Table 2), attesting to the reliability of the data subsequently analyzed. Analysis of variance (ANOVA) was used to demonstrate significant differences between the groups of basketball players. On the other hand, ANCOVA analysis was employed to show differences when considering maturity timing, using chronological age, body height, and mass used as covariates. Bonferroni adjustments were made for post-hoc comparisons. The effect size was determined using partial eta squared (η^2^) and was classified as follows: no effect = 0 to 0.039, minimum = 0.04 to 0.24, moderate = 0.25 to 0.63, and strong =  ≥ 0.64^59^.

**Table 2:** Cronbach’s α statistic and percentage of interrater reliability for the basic anthropometric variables and physical tests.

Variable	α	% Agreement (%)	95% CI
5 m	0.914	91	0.902–0.928
10 m	0.923	92	0.910–0.933
20 m	0.916	91	0.901–0.925
Agility—S_1_	0.931	93	0.923–0.948
Agility—S_2_	0.940	94	0.930–0.950
Agility—S_3_	0.934	93	0.921–0.941
Agility—S_4_	0.943	94	0.932–0.954
Agility—S_5_	0.939	94	0.930–0.951
Agility_Total_	0.941	94	0.932–0.954
SVJ_max_	0.963	96	0.951–0.973
VJ_max_	0.952	95	0.944–0.965
SVJ	0.892	89	0.881–0.903
VJ	0.908	90	0.892–0.913
Yo-Yo distance	0.917	91	0.901–0.924
Yo-Yo VO_2max_	0.920	92	0.910–0.930

*S*_*1*_*–S*_*5*_ stages, *SVJ* standing vertical jump, *VJ* vertical jump.

Backward stepwise multiple regression was used to estimate the relative contributions of chronological age, maturity timing (stage of APHV), body height, body mass, and height x mass interaction (based on residuals) to the variability in individual physical fitness tests. In all the analyses, the significance of the effects was set at *p* < 0.05. All calculations were performed using STATISTICA software (v.13.3, StatSoft, USA).

### Ethics approval

The research was conducted in accordance with the approval from the local Ethics Committee for Scientific Research of the University of Physical Education in Warsaw (SKE 01–28/2016), and the study was completed according to the rules and regulations of the Declaration of Helsinki.

### Consent to participate

Informed consent was obtained from the parents or legal guardians of the participants.

All of the participants were informed in writing about the aims, benefits, and procedures of the research project, as well as the possibility to withdraw from the study at any moment without providing an explanation. The inclusion criterion was the written informed consent of each participant, and the exclusion criteria included contraindications for the basic anthropometric measurements. Any injury or trauma also caused exclusion from the study.

## Results

Table 1 shows the distribution of the studied basketball players with respect to chronological age and maturity timing (Table 1). The presence of early and average maturers among basketball players was observed in all three groups: 48.5% and 51.5% in 13 year olds, 51.1% and 48.9% in 14 year olds, and 41.2%, and 48.8% in 15 year olds, respectively. No late-maturing basketball players were found.

When analysing the variation of basketball players with respect to chronological age (Table 3), significantly (p < 0.001) lower body height (by 4.2% and 6.7%, respectively; F_(2,815)_ = 110.32; η^2^ = 0.21, minimum effect), body mass (by 11.5% and 19%, respectively; F_(2,815)_ = 74.67; η^2^ = 0.15, minimum effect), and standing reach (by 4.1% and 6.4%, respectively; F_(2,815)_ = 92.39; η^2^ = 0.18, minimum effect) were observed in the under 13-year-old group compared to the under 14- and 15-year-old groups. Furthermore, under 14-year-old basketball players were characterized by lower values (p < 0.001) of body height (by 2.6%), body mass (by 8.5%), and standing reach (by 2.4%) compared to those under 15 years of age.

**Table 3:** Descriptive statistics of male basketball players based on chronological age and ANOVA results comparing age groups.

Variable	Under 13 (n = 233)		Under 14 (n = 364)		Under 15 (n = 221)		*F (p)*	*η2*	*d*
	M	SEM	M	SEM	M	SEM			
Chronological age (years)	13.04	0.02	13.96	0.02	14.75	0.02	–	–	–
APHV (years)	13.11	0.03	13.10	0.02	13.15	0.03	1.23	< 0.01	
Body height (cm)	168.3	0.6	175.7	0.5	180.4	0.5	110.32 (**)	0.21	1v2v3
Body mass (kg)	56.3	0.8	63.6	0.6	69.5	0.8	74.67 (**)	0.15	1v2v3
Standing reach (cm)	226.1	0.9	235.8	0.6	241.6	0.7	92.39 (**)	0.18	1v2v3
5 m (s)	1.177	0.007	1.141	0.005	1.134	0.006	13.86 (**)	0.04	1v2,3
10 m (s)	2.001	0.010	1.929	0.007	1.905	0.007	36.50 (**)	0.08	1v2,3
20 m (s)	3.488	0.016	3.333	0.010	3.262	0.011	74.88 (**)	0.15	1v2v3
Agility—S_1_ (s)	1.591	0.011	1.555	0.009	1.553	0.009	4.44 (*)	0.04	1v2,3
Agility—S_2_ (s)	5.495	0.047	5.346	0.026	5.179	0.038	16.27 (**)	0.04	1v2v3
Agility—S_3_ (s)	7.387	0.050	7.108	0.032	6.934	0.038	28.83 (**)	0.07	1v2v3
Agility—S_4_ (s)	9.668	0.062	9.296	0.045	9.086	0.048	27.69 (**)	0.06	1v2v3
Agility—S_5_ (s)	13.148	0.082	12.606	0.055	12.288	0.060	37.47 (**)	0.08	1v2v3
Agility_Total_ (s)	15.052	0.094	14.429	0.063	14.063	0.067	38.46 (**)	0.09	1v2v3
SVJ_max_ (cm)	268.9	1.1	283.1	0.8	292.1	0.8	146.28 (**)	0.26	1v2v3
VJ_max_ (cm)	280.5	1.2	296.0	0.9	307.4	1.0	149.59 (**)	0.27	1v2v3
SVJ (cm)	42.8	0.5	47.3	0.4	50.6	0.5	65.03 (**)	0.14	1v2v3
VJ (cm)	54.4	0.7	60.2	0.5	65.9	0.6	77.96 (**)	0.16	1v2v3
Yo-Yo distance (m)	1003	33	1269	29	1423	40	34.04 (**)	0.08	1v2v3
Yo-Yo VO_2max_ (ml/kg/min)	44.8	0.3	47.1	0.2	48.4	0.3	33.99 (**)	0.08	1v2v3

*M* mean, *SEM* standard error of mean, *S*_*1*_*–S*_*5*_ stages, *SVJ* standing vertical jump, *VJ* vertical jump. *d *significant differences between groups (1—under 13; 2—under 14, 3—under 15).

*p < 0.05, **p < 0.001.

In the sprint test over all distances, under 13-year-old players were slower (p < 0.001) than those from the under 14-year-old and 15-year-old groups (within 3–7%, minimum effect, respectively). However, the comparison between 14- and 15-year-old players showed no significant differences in the sprinting time at distances of 5 m and 10 m. For the 20-m test, the times achieved by 14-year-old players were slower than those achieved by 15-year-old players by 2.8% (F_(2,815)_ = 74.88; η^2^ = 0.15, minimum effect).

Analysis of the results of the agility test results revealed that at all distances, under 13-year-old players were slower than under 14- and 15-year-old players (2–7%, minimum effect), and 14-year-old players were slower compared to under 15-year-old players (2–4%, minimum effect), except for stage 1 (S_1_), where no significant differences were observed between under 14- and 15-year-old players. Analysis of the results in the context of the jumping tests revealed significantly (p < 0.001) lower values (7–18%; moderate effect in SVJ_max_ and VJ_max_; minimum effect in SVJ and VJ) obtained by players under 13 years compared to players under 14 and 15 years of age. An identical relationship (lower values within 3–9%) was observed in players under 14 years compared to those under 15 years.

Basketball players from the under 13-year-old group compared to players under 14- and 15-years-old were also characterized by significantly (p < 0.001) lower values of the distance covered (21–30%, F_(2,815)_ = 34.04; η^2^ = 0.08, minimum effect) and VO_2_max (4–8%, F_(2,815)_ = 33.99; η^2^ = 0.08; minimum effect) in the physical capacity test. Additionally, athletes under 14 years old compared to athletes under 15 years old covered 10.8% less distance and had 2.7% lower VO_2_max values.

Table 4 shows the age-adjusted characteristics of basketball players with respect to maturity timing (Table 4). Except for S_1_ in the agility test, the under 13-year-old basketball players showed significant differences in favor of the early maturers in all motor variables tested. For anthropometric traits, the differences were 9–31%, whereas differences of 2 to 17% were observed for physical fitness tests in favor of the early maturers. In the group of early maturing players under 14 years, significantly higher values of basic anthropometric traits (p < 0.001, 7–26%), 20-m speed (by 1.4%), and jumping ability (3–7%) and significantly lower values in the endurance test (within 1–2%) were reported. Furthermore, early maturing players under 15 years compared to middle maturers were taller (by 6.8%) and heavier (by 22.1%) and had higher values in the jumping test (within 1–6%). In contrast, early maturers from the under 15-year-old group obtained lower values in the endurance test (1–8%).

**Table 4:** Age-adjusted means (standard errors are given in parentheses) by stage of maturity within age groups and ANCOVA results with decimal age as a covariate.

Variable	Under 13		*F(p)*	*η*^*2*^	Under 14		*F(p)*	*η*^*2*^	Under 15		*F(p)*	*η*^*2*^
	Early (n = 113)	Average (n = 120)			Early (n = 186)	Average (n = 178)			Early (n = 91)	Average (n = 130)		
Body height (cm)	176.1 (0.5)	161.0 (0.5)	55.84 (***)	0.20	182.4 (0.4)	168.7 (0.4)	29.4 (***)	0.14	187.4 (0.5)	175.4 (0.4)	7.40 (***)	0.06
Body mass (kg)	64.1 (0.8)	49.0 (0.8)	3.64 (*)	0.03	70.7 (0.6)	56.2 (0.7)	10.3 (***)	0.05	77.8 (0.9)	63.7 (0.8)	7.84 (***)	0.07
Standing reach (cm)	236.7 (0.8)	216.0 (0.7)	22.30 (***)	0.16	244.3 (0.6)	226.9 (0.6)	21.7 (***)	0.11	250.4 (0.8)	235.4 (0.6)	0.55 (ns)	–
5 m (s)	1.166 (0.010)	1.189 (0.010)	5.15 (**)	0.04	1.132 (0.007)	1.149 (0.007)	0.18 (ns)	–	1.123 (0.009)	1.141 (0.008)	0.22 (ns)	–
10 m (s)	1.979 (0.013)	2.023 (0.013)	7.69 (**)	0.06	1.916 (0.009)	1.942 (0.009)	0.90 (ns)	–	1.899 (0.011)	1.909 (0.009)	0.06 (ns)	–
20 m (s)	3.445 (0.022)	3.531 (0.021)	9.77 (**)	0.08	3.310 (0.014)	3.357 (0.014)	3.52 (*)	0.02	3.253 (0.017)	3.268 (0.015)	1.91 (ns)	–
Agility—S_1_ (s)	1.563 (0.015)	1.618 (0.015	0.89 (ns)	–	1.537 (0.012)	1.573 (0.013)	1.31 (ns)	–	1.533 (0.013)	1.568 (0.011)	0.29 (ns)	–
Agility—S_2_ (s)	5.362 (0.066)	5.631 (0.064)	4.92 (**)	0.04	5.342 (0.037)	5.350 (0.037)	3.20 (ns)	–	5.130 (0.060)	5.221 (0.050)	1.52 (ns)	–
Agility—S_3_ (s)	7.233 (0.068)	7.538 (0.066)	8.33 (**)	0.07	7.104 (0.044)	7.112 (0.045)	3.04 (ns)	–	6.894 (0.059)	6.968 (0.050)	1.09 (ns)	–
Agility—S_4_ (s)	9.548 (0.088)	9.784 (0.085)	5.57 (**)	0.05	9.352 (0.062)	9.238 (0.064)	2.57 (ns)	–	9.031 (0.075)	9.127 (0.063)	0.62 (ns)	–
Agility—S_5_ (s)	12.954 (0.113)	13.347 (0.110)	8.12 (**)	0.07	12.625 (0.077)	12.586 (0.079)	2.91 (ns)	–	12.209 (0.094)	12.350 (0.079)	1.70 (ns)	–
Agility_Total_ (s)	14.837 (0.129)	15.274 (0.125)	8.79 (**)	0.07	14.451 (0.088)	14.406 (0.090)	2.31 (ns)	–	14.002 (0.104)	14.113 (0.087)	1.62 (ns)	–
SVJ_max_ (cm)	280.6 (1.0)	257.6 (0.9)	27.18 (***)	0.19	292.1 (0.8)	273.7 (0.8)	26.86 (***)	0.13	301.4 (1.0)	285.6 (0.9)	3.85 (*)	0.03
VJ_max_ (cm)	293.0 (1.1)	268.5 (1.0)	33.71 (***)	0.23	305.6 (0.9)	286.0 (1.0)	23.93 (***)	0.12	316.9 (1.2)	300.7 (1.0)	4.60 (*)	0.04
SVJ (cm)	43.9 (0.7)	41.6 (0.7)	4.19 (*)	0.03	47.8 (0.5)	46.8 (0.5)	5.10 (**)	0.03	51.0 (0.7)	50.2 (0.6)	4.12 (*)	0.04
VJ (cm)	56.3 (0.9)	52.5 (0.9)	9.84 (**)	0.08	61.2 (0.7)	59.1 (0.7)	5.83 (**)	0.03	66.5 (0.9)	65.5 (0.8)	5.48 (**)	0.05
Yo-Yo distance (m)	1081 (47)	927 (46)	3.15 (*)	0.03	1240 (40)	1300 (41)	5.24 (**)	0.03	1359 (62)	1465 (51)	3.15 (*)	0.03
Yo-Yo VO_2max_ (ml/kg/min)	45.5 (0.4)	44.2 (0.4)	3.13 (*)	0.03	46.8 (0.3)	47.3 (0.3)	5.24 (**)	0.03	47.8 (0.5)	48.7 (0.4)	3.15 (*)	0.03

*S*_*1*_*–S*_*5*_ stages, *SVJ* standing vertical jump, *VJ* vertical jump, *ns* nonsignificant difference.

*p < 0.05, **p < 0.01, ***p < 0.001.

ANCOVA analysis (decimal age, body height, and body mass as a covariate) when considering the maturity timing (Table 5) showed that early maturing basketball players under 13 years of age achieved significantly better results in starting speed (by 3.5%; p < 0.05; F_(7,225)_ = 3.73; η^2^ = 0.03, no effect), the 10-m test (by 2.9%; p < 0.01; F_(7,225)_ = 5.13; η^2^ = 0.04, minimum effect), and the 20-m test (by 2.3%; p < 0.01; F_(7,225)_ = 5.26; η^2^ = 0.04, minimum effect). A similar trend was found in stages 2–5 (S_2_ -S_5_) and total time in the agility trial, where the early maturers were faster by 3–9%. Additionally, early maturing players under 13 years of age had higher values in all tests of jumping ability (1–9%) and endurance (3–22%) compared to average maturers. In contrast, compared to average maturing peers, early maturing basketball players under 14 years of age (Table 5) had significantly lower values of running vertical jump (by 2.5%; p < 0.001; F_(7,356)_ = 9.87; η^2^ = 0.05, minimum effect), distance covered (by 4%; p < 0.01; F_(7,356)_ = 4.87; η^2^ = 0.03, no effect) and VO_2_max (by 1%; p < 0.01; F_(7,356)_ = 4.88; η^2^ = 0.03, no effect) in the endurance test. An identical trend was observed between early- and mid-maturers from the group of under 15-year-old basketball players, with the former showing poorer performance (within 1–4%) in the running vertical jump and endurance tests.

**Table 5:** Age-adjusted means (standard errors are given in parentheses) by stage of maturity within age groups and ANCOVA results with decimal age, body height, and body mass as covariates.

Variable	Under 13		*F(p)*	*η*^*2*^	Under 14		*F(p)*	*η*^*2*^	Under 15		*F(p)*	*η*^*2*^
	Early (n = 113)	Average (n = 120)			Early (n = 186)	Average (n = 178)			Early (n = 91)	Average (n = 130)		
Standing reach (cm)	226.7 (0.7)	226.8 (0.7)	0.35 (ns)	–	236.3 (0.7)	235.4 (0.7)	2.03 (ns)	–	243.0 (0.8)	243.5 (0.7)	0.88 (ns)	–
5 m (s)	1.159 (0.017)	1.200 (0.017)	3.73 (*)	0.03	1.150 (0.011)	1.136 (0.012)	0.50 (ns)	–	1.124 (0.010)	1.141 (0.008)	0.50 (ns)	–
10 m (s)	1.972 (0.022)	2.030 (0.023)	5.13 (**)	0.04	1.931 (0.015)	1.912 (0.015)	0.75 (ns)	–	1.892 (0.020)	1.895 (0.015)	0.20 (ns)	–
20 m (s)	3.453 (0.036)	3.534 (0.036)	5.26 (**)	0.04	3.321 (0.024)	3.286 (0.023)	0.42 (ns)	–	3.234 (0.031)	3.234 (0.024)	0.09 (ns)	–
Agility—S_1_ (s)	1.617 (0.026)	1.607 (0.026)	0.15 (ns)	–	1.551 (0.021)	1.563 (0.021)	0.20 (ns)	–	1.542 (0.024)	1.560 (0.019)	1.38 (ns)	–
Agility—S_2_ (s)	5.236 (0.113)	5.712 (0.115)	4.68 (*)	0.04	5.333 (0.061)	5.272 (0.061)	0.60 (ns)	–	5.213 (0.107)	5.187 (0.084)	1.69 (ns)	–
Agility—S_3_ (s)	7.087 (0.115)	7.526 (0.117)	6.99 (**)	0.06	7.072 (0.075)	7.013 (0.074)	0.70 (ns)	–	6.861 (0.107)	6.945 (0.084)	1.25 (ns)	–
Agility—S_4_ (s)	9.402 (0.149)	9.781 (0.152)	4.67 (*)	0.04	9.241 (0.105)	9.141 (0.104)	2.36 (ns)	–	8.929 (0.136)	9.065 (0.107)	0.70 (ns)	–
Agility—S_5_ (s)	12.778 (0.191)	13.336 (0.195)	6.26 (**)	0.05	12.528 (0.130)	12.410 (0.129)	1.93 (ns)	–	12.095 (0.169)	12.272 (0.133)	1.22 (ns)	–
Agility_Total_ (s)	14.672 (0.217)	15.247 (0.221)	6.37 (**)	0.05	14.341 (0.148)	14.191 (0.146)	2.02 (ns)	–	13.840 (0.188)	14.021 (0.148)	1.41 (ns)	–
SVJ_max_ (cm)	270.8 (1.3)	267.7 (1.3)	3.46 (*)	0.03	285.0 (1.0)	284.5 (1.0)	1.30 (ns)	–	293.1 (1.4)	294.3 (1.1)	0.86 (ns)	–
VJ_max_ (cm)	284.1 (1.5)	280.0 (1.5)	6.94 (**)	0.06	297.2 (1.3)	297.7 (1.2)	0.70 (ns)	–	309.7 (1.7)	312.2 (1.3)	2.54 (ns)	–
SVJ (cm)	44.1 (1.2)	40.8 (1.2)	5.13 (*)	0.04	48.7 (0.9)	49.1 (0.9)	0.36 (ns)	–	50.1 (1.3)	50.8 (1.0)	1.94 (ns)	–
VJ (cm)	57.4 (1.5)	53.1 (1.5)	5.89 (**)	0.05	60.9 (1.2)	62.3 (1.2)	9.87 (***)	0.05	66.7 (1.6)	68.9 (1.2)	7.54 (**)	0.07
Yo-Yo distance (m)	1146 (78)	939 (80)	3.14 (*)	0.03	1334 (67)	1389 (66)	4.87 (**)	0.03	1374 (110)	1437 (87)	3.87 (*)	0.03
Yo-Yo VO_2max_ (ml/kg/min)	46.0 (0.7)	44.3 (0.7)	3.13 (*)	0.03	47.6 (0.6)	48.1 (0.6)	4.88 (**)	0.03	47.9 (0.9)	48.5 (0.7)	3.86 (*)	0.03

*S*_*1*_*–S*_*5*_ stages, *SVJ* standing vertical jump, *VJ* vertical jump, *ns* nonsignificant difference.

*p < 0.05, **p < 0.01, ***p < 0.001.

The results of the backward stepwise multiple regression analysis are presented in Table 6. The presented model explained 8–25% (adjusted *R*^*2*^ = 0.08–0.25; *p* < 0.001) of the variance in individual strength and conditioning tests. Maturity timing was a significant predictor (negative value of the standardized β coefficient) for VJ (adjusted *R*^*2*^ = 0.20; *p* < 0.001) and VO_2_max (adjusted *R*^*2*^ = 0.10; *p* < 0.001) tests. Furthermore, body height was a significant predictor for 5-m (adjusted *R*^*2*^ = 0.08; *p* < 0.001), 10-m (adjusted *R*^*2*^ = 0.15; *p* < 0.001), 20-m trial (adjusted *R*^*2*^ = 0.25; *p* < 0.001) and agility (negative direction of β coefficient; adjusted *R*^*2*^ = 0.14; *p* < 0.001)), as well as SVJ (adjusted *R*^*2*^ = 0.20; *p* < 0.001), VJ, and VO_2_max tests (positive direction of β coefficient). The predictor of interactions of body height and mass was significant for 10-m speed, 20-m speed, agility (positive direction), and SVJ and VJ tests (negative direction). Body mass itself was a significant predictor only in the 5-m trial. It was also found that chronological age was a significant predictor in the speed test (negative direction), and agility and endurance tests (additive direction).

**Table 6:** Summary of the backward regression for motor skills and anthropometric variables by the male basketball players aged 13–15 years.

Attempt	Predictor	Standardized *β*	Adjusted *R*^2^	*F (p)*
5 m	Body height	−0.421	0.08	30.32 (*)
	Body mass	0.280		
	Chronological age	−0.225		
10 m	Body height	−0.508	0.15	48.81 (*)
	Mass*height interaction	0.402		
	Chronological age	−0.335		
20 m	Body height	−0.551	0.25	91.62 (*)
	Mass*height interaction	0.422		
	Chronological age	−0.298		
Agility_Total_	Body height	−0.365	0.14	45.29 (*)
	Mass*height interaction	0.336		
	Chronological age	0.354		
SVJ	Body height	0.370	0.20	66.91 (*)
	Mass*height interaction	−0.299		
VJ	Maturity	−0.268	0.20	69.60 (*)
	Body height	0.841		
	Mass*height interaction	−0.347		
Yo-Yo VO_2max_	Maturity	−0.222	0.10	30.25 (*)
	Chronological age	0.272		
	Body height	0.553		

*SVJ* standing vertical jump, *VJ* vertical jump;

*p < 0.001.

## Discussion

The aims of this study were (i) to identify the motor potential and basic anthropometric characteristics of Polish basketball players aged 13 to 15 years, (ii) to demonstrate the effect of maturity timing on the results achieved in motor tests and basic body composition parameters and (iii) to determine which index contributes most to the prediction of performance in the individual tests of speed, jumping ability, agility, and endurance.

The results of this study are consistent with those obtained in similar studies^60^, where no late maturers were reported in youth football academies. This finding is likely to be due to the qualification for the sport made by coaches during this training stage^34, 37^. Late maturers are rarely considered in the training process. During recruitment and selection at the youth level, club coaches need to consider that the advantage in body size and physical performance of older and early-maturing players may confound the potential assessment of the athlete and result in talent loss^61^. Very often, players with an advanced maturity status outperform their late-maturing peers in static strength, endurance, sprint, agility, jumps, and throwing tasks^25, 36, 37^. However, the maturational benefits decrease with age as the differences in biological maturity become less pronounced and completely diminish after full adult status^62^.

This finding could explain the teams' better results in youth competitions given that most of the players were early maturing players. Very often, such training groups undergo early specialization, and athletes with potential cannot fully develop. This notion is consistent with a study by Ribeiro Junior et al.^21^, where players with potential (who were not selected early for national teams), without early specialization due to playing position were more likely to reach the professional level of competition.

Without considering the biological status in all anthropometric traits and motor tests, older basketball players achieved higher values and better results. Considering only the chronological age of Polish basketball players, the results in individual age categories were comparable to other such studies^25, 31, 63^, emphasizing the importance of anthropometric (i.e., body height, body weight, and arm span and reach) and functional (i.e., speed, agility, upper body strength, and jumping ability) characteristics of performance in young players^26, 27, 64^. Polish basketball players under 13 years score slightly (by 5%; n = 16) lower body height values than their selected Portuguese peers^25^ and were taller (by 3.2%; n = 134) than nonselected individuals. On the other hand, Polish athletes under 14 and 15 achieve better results in basic anthropometric parameters than their peers from Portugal (within 1–2%; n = 31 and n = 28, respectively)^31^.

Regarding the results of individual strength and conditioning tests, Polish under 14-year-old basketball players had better (within 6%) results in the 20-m speed test compared to their peers from Serbia (n = 50)^65^ and Portugal (n = 55)^64^ even when taking into account their maturity timing. In the same test, under 13-year-old players from Poland obtained better (by 8%) results when considering biological maturity compared to Serbian basketball players (n = 48)^40^ and almost identical results to Slovenian players (n = 86)^32^. However, these players were 4% slower than Italian basketball players (n = 50)^66^. Furthermore, the Polish U15 group had better results (when considering maturity timing) in the 10-m and 20-m speed tests (by 10% and 11%, respectively) compared to the Greek basketball players (n = 23)^67^. However, in the jump test, Polish basketball players under 14 and under 15 years old were characterized by lower VJ values (by 14.6% and 13% respectively) than their peers from the Netherlands (n = 48)^39^. The same trend was found when considering the maturity timing. The comparison of endurance of Polish and Portuguese under 14-year-old basketball players (n = 55)^64^ revealed higher values (6–12%) in Polish basketball players, even when taking into account their maturity timing. Early maturing players tend to have better performance in speed, agility, and lower limb strength and a higher likelihood of being selected by coaches to train and play with top athletes^68^. It is important to mention that varied and nonspecific early involvement in sports may have a beneficial effect on jumping and sprinting performance^69^.

In the case of the values related to age-adjusted averages of Polish basketball players (Table 4), it was observed that in the under 13-year-old group, early maturers had significantly better results (except for S_1_ in the agility test) than average maturers. However, in the endurance test in the under 14- and 15-year-old groups (both distance covered and VO_2max_), the average maturers obtained higher values. Furthermore, maturity differentiation in the under 14- and 15-year-old groups significantly affected body size, 20-m speed (under 14 only), and the results of all jumping tests. However, no differences in agility and speed at distances of 5-m and 10-m were observed. This finding is consistent with previous studies in the same field (n = 59)^31^, which have attributed this situation to the overrepresentativeness of early and normal maturers in sports groups in the under 13- and 14-year-old categories. Late maturing boys are overlooked during the selection process or withdraw from the sport with the increasing demands of specialization. Therefore, Coelho e Silva et al.^31^ proposed extending the selection process to at least closer to the end of the adolescent growth phase and developing dedicated training programs with content tailored to late maturers.

Analysis of means adjusted for age, body height, and body mass (Table 5) showed better results of early maturers in the under 13-year-old group. In contrast, the opposite trend was observed in the under 14- to 15-year-old groups, where early maturing individuals performed worse in the running vertical jump (VJ) and endurance tests (both distances covered and VO_2_max). No significant differences were noted between the other parameters. This finding may serve as evidence that in these groups, the advantage in motor abilities, resulting from the characteristics of body build and proportions (body height, body mass) and their changes correlated with the processes of biological maturation, begins to disappear at the age of 14 years^31, 70^. A further implication of the trend is that adolescent basketball players and perhaps athletes in other sports should be grouped based on biological maturity and variations in chronological age^31^.

Age, body height, body mass, mass-height interaction, and biological status accounted for 8–25% of the variance in individual physical fitness tests (Table 6). Maturity timing was a significant predictor only in VJ and VO_2_max, but body height was the most significant predictor in these tests. This finding is supported by previous reports (n = 37), where biological maturity (estimated as a percentage of predicted adult height) was a strong predictor of VO_2_max^71^. In another study (n = 13) somatic maturation (estimated as a percentage of predicted adult height) predicted exercise response in lower-body repeated power ability (RPA)^33^. The results indicated that lower-body RPA implies higher cardio-respiratory performance (e.g. the mean oxygen uptake)^33^. The regression results for VJ and VO_2_max (maturity timing: direction and value) and the adjusted values for decimal age, body height, and body mass (where the mean values of average maturers from 14-year-old and 15-year-old groups were slightly higher than in the early maturing peers) might be explained as a response to a combination of anatomical changes occurring in the structure, size, metabolism, and the neuromuscular system^72, 73^. These changes determine the ability of the cardiopulmonary system to cope with increased training demands in the later stages of puberty^73^. This may also result from previous training experiences in other sports, which was confirmed by Arede et al.^69^ who showed that more specialized athletes showed less neuromuscular functions efficiency (jumping performance) than less specialized basketball players who trained versatile sports at the early stages of life (at the age of 6–10 years). The results highlight the importance of considering the variability associated with biological maturation in the aerobic capacity of boys in late puberty^33^.

One limitation of the study is the lack of a comprehensive explanation of the effect of maturity timing on VO_2_max through body components (e.g., lean body mass), which were not studied. The second limitation is that the Yo-Yo IR1 protocol was not previously validated for basketball youth athletes at that age^74^. All equations used to predict YAPHV (maturity shift) or APHV have the same major limitations^75, 76^. The advanced maturity timing of adolescent male athletes and the relatively narrow range of variation in predicted age at peak height velocity (PHV) may undermine its utility and effectiveness in talent identification and development programs when applied at a specific time point^29, 38^. Although the stability of predictions within individuals is poor and group classification is not accurate, APHV predicted by the Mirwald equation^23^ can be used successfully among boys who reach average (in time) maturation and in the period of rapid growth (12–15 years)^38^. Given the importance of biological maturity in talent identification and the difficulties in implementing maturity protocols other than those mentioned above, the resulting data covering a large sample of Polish players can help coaches identify individuals with the best potential. The results are thus specific to Polish basketball players of Caucasian ancestry. Generalization to players from other ethnicities needs to be done with care.

Despite the limitations in using equations to predict YAPHV^75, 76^, further research into Polish young basketball players in terms of talent identification taking into account biological maturation in correlation to a player's transfer from youth categories to the professional senior league is needed. As in the case of the study of Brazilian basketball players (where only 10% of players participating in the youth championships in Brazil reached the professional league)^21^ and Spanish players (a small percentage of athletes considered talented from youth national teams achieved excellent senior results)^77^, the career progression of Polish basketball players should be verified in additional studies. Further research on the influence of biological maturity on the performance of Polish and foreign basketball players (e.g., places in national championships) and participation in a specific level of competition should be performed. These studies should also consider training volume and intensity, body components, perceptual-cognitive elements, and tactical skills.

## Conclusion

In the group of under 13-year-old basketball players, maturity timing especially influences performance during physical fitness tests. However, in the under 14- and 15-year-old groups, physical fitness tests results (after controlling for variations in decimal age, body height, and body mass) showed a tendency to reduce the differences between early and average maturing players (except for VJ and endurance tests: both distances covered and VO_2_max), with average maturers achieving better results. Maturity timing (VJ and VO_2_max), chronological age (5 m, 10 m, 20 m, agility, and VO_2_max tests), body height (all tests), body mass (5 m), and interaction of body mass and height (10 m, 20 m, agility, SVJ, VJ) were significant predictors of motor skills. In the practice of the training process, these findings can be helpful in quantifying and controlling the results of motor programs adjusted to biological requirements. The findings should also encourage coaches to be more patient in the process of developing young talents and to give players time to develop to reach their full potential.

## Data Availability

Full access to data on request (karol.gryko@awf.edu.pl).

## References

[1] Muratovic A, Vujovic D, Hadzic R (2014). Comparative study of anthropometric measurement and body composition between elite handball and basketball players. Monten. J. Sports Sci. Med..

[2] Kostopoulos N (2015). Anthropometric and fitness profiles of young basketball players according to their playing position and time. J. Phys. Educ. Sport..

[3] Stojanović E, Aksović N, Stojiljković N, Stanković R, Scanlan AT, Milanović Z (2019). Reliability, usefulness, and factorial validity of change-of-direction speed tests in adolescent basketball players. J. Strength Cond. Res..

[4] Stojanović E, Stojiljković N, Scanlan AT, Dalbo VJ, Berkelmans DM, Milanović Z (2018). The activity demands and physiological responses encountered during basketball match-play: A systematic review. Sports Med..

[5] Meckell Y, Casorla T, Eliakim A (2009). The influence of basketball dribbling on repeated sprints. Int. J. Coach. Sci..

[6] Gryko K, Mikołajec K, Maszczyk A, Cao R, Adamczyk JG (2018). Structural analysis of shooting performance in elite basketball players during FIBA EuroBasket 2015. Int. J. Perform. Anal. Sport..

[7] Hoover SJ, Winner RK, McCutchan H, Beaudoin CC, Judge LW, Jones LM, Leitzelar B, Hoover DL (2017). Mood and performance anxiety in high school basketball players: A pilot study. Int. J. Exerc. Sci..

[8] Edwards T, Spiteri T, Piggott B, Bonhotal J, Haff GG, Joyce C (2018). Monitoring and managing fatigue in basketball. Sports (Basel, Switzerland)..

[9] Castagna, C., Impellizzeri, F.M., Chaouachi, A., Ben Abdelkrim, N. & Manzi, V. Physiological responses to ball-drills in regional level male basketball players. *J. Sports Sci*. **29(12)**, 1329–1336 (2011). 10.1080/02640414.2011.597418 21777056

[10] Ziv G, Lindor R (2009). Physical attributes, physiological characteristics, on-court performances and nutritional strategies of female and male basketball players. Sports Med..

[11] Scanlan AT, Dascombe BJ, Reaburn PR (2012). The construct and longitudinal validity of the basketball exercise simulation test. J. Strength Cond. Res..

[12] Wen N, Dalbo VJ, Burgos B, Pyne DB, Scanlan AT (2018). Power testing in basketball: Current practice and future recommendations. J Strength Cond. Res..

[13] Scanlan AT, Tucker PS, Dalbo VJ (2014). A comparison of linear speed, closed-skill agility, and open-skill agility qualities between backcourt and front- court adult semiprofessional male basketball players. J. Strength Cond. Res..

[14] Johnson, S.R. *et al.* A coach's responsibility: Learning how to prepare athletes for peak performance. *Sport J*. **14** (2011).

[15] Barreiros A, Côté J, Fonseca AM (2014). From early to adult sport success: Analysing athletes’ progression in national squads. Eur. J. Sport Sci..

[16] Bompa, T.O. *Total Training for Young Champions. Human Kinetics* (Champaign, 2000).

[17] Malina RM, Bouchard C, Bar-Or O (2004). Growth, Maturation, and Physical Activity.

[18] Myburgh GK, Cumming SP, Malina RM (2019). Cross-sectional analysis investigating the concordance of maturity status classifications in elite caucasian youth tennis players. Sports Med Open..

[19] Malina RM, Cumming SP, Kontos AP, Eisenmann JC, Ribeiro B, Aroso J (2005). Maturity-associated variation in sport-specific skills of youth soccer players aged 13–15 years. J. Sports Sci..

[20] Baker, J. & Wattie, N. Innate talent in sport: separating myth from reality. *Curr. Issues Sport Sci*. **3**(6). 10.36950/2018ciss006 (2018)

[21] Ribeiro Junior DB, Werneck FZ, Oliveira HZ, Panza PS, Ibáñez SJ, Vianna JM (2021). From talent identification to Novo Basquete Brasil (NBB): Multifactorial analysis of the career progression in youth Brazilian elite basketball. Front. Psychol..

[22] Kozieł S, Malina RM (2018). Modified maturity offset prediction equations: Validation in independent longitudinal samples of boys and girls. Sports Med..

[23] Mirwald RL, Baxter-Jones ADG, Bailey DA, Beunen GP (2002). An assessment of maturity from anthropometric measurements. Med. Sci. Sports Exerc..

[24] Santos EJ, Janeira MA (2011). The effects of plyometric training followed by detraining and reduced training periods on explosive strength in adolescent male basketball players. J. Strength Cond. Res..

[25] Guimarães E, Baxter-Jones A, Maia J, Fonseca P, Santos A, Santos E, Tavares F, Janeira MA (2019). The roles of growth, maturation, physical fitness, and technical skills on selection for a Portuguese under-14 years basketball team. Sports..

[26] Torres-Unda J, Zarrazquin I, Gil J, Ruiz F, Irazusta A, Kortajarena M, Seco J, Irazusta J (2013). Anthropometric, physiological and maturational characteristics in selected elite and non-elite male adolescent basketball players. J. Sports Sci..

[27] Torres-Unda J, Zarrazquin I, Gravina L, Zubero J, Seco J, Gil S, Gil J, Irazusta J (2016). Basketball performance is related to maturity and relative age in elite adolescent players. J. Strength Cond. Res..

[28] Arede J, Cumming S, Leite N (2019). Does bio-banding influence physical performance profile in youth basketball?. J. Sports Sci..

[29] Arede J, Fernandes J, Moran J, Norris J, Leite N (2021). Maturity timing and performance in a youth national basketball team: Do early-maturing players dominate?. Int. J. Sports Sci. Coach..

[30] Guimarães E, Baxter-Jones ADG, Williams AM, Tavares F, Janeira MA, Maia J (2021). The role of growth, maturation and sporting environment on the development of performance and technical and tactical skills in youth basketball players: The INEX study. J. Sports Sci..

[31] Coelho e Silva, M.J., Figueiredo, A.J., Carvalho, H.M. & Malina, R.M. Functional capacities and sport-specific skills of 14- to 15-year-old male basketball players: Size and maturity effects. *Eur. J. Sport Sci*. **8**, 277–285 (2008).

[32] Štrumbelj E, Erčulj F (2014). Analysis of experts’ quantitative assessment of adolescent basketball players and the role of anthropometric and physiological attributes. J. Hum. Kinet..

[33] Arede, J., Leite, N., Bradley, B., Madruga-Parera, M., Saéz de Villarreal, E., & Gonzalo-Skok, O. Mechanical, physiological, and perceptual demands of repeated power ability lower-body and upper-body tests in youth athletes: Somatic maturation as a factor on the performance. *Front. Psychol.* **11**, 1888 (2020). 10.3389/fpsyg.2020.01888 10.3389/fpsyg.2020.01888 PMC7411083 32849108

[34] te Wierike SC, Elferink-Gemser MT, Tromp EJ, Vaeyens R, Visscher C (2015). Role of maturity timing in selection procedures and in the specialisation of playing positions in youth basketball. J. Sports Sci..

[35] Carvalho HM, Silva M, Figueiredo AJ, Gonçalves CE, Phillippaerts RM, Castagna C, Malina RM (2011). Predictors of maximal short-term power outputs in basketball players 14–16 years. Eur. J. Appl. Physiol..

[36] Carvalho HM, Gonçalves CE, Collins D, Paes RR (2018). Growth, functional capacities and motivation for achievement and competitiveness in youth basketball: An interdisciplinary approach. J. Sports Sci..

[37] Arede J, Ferreira AP, Gonzalo-Skok O, Leite N (2018). Maturational development as key aspect in physical performance and national team selection in elite male basketball players. Int. J. Sports Physiol. Perform..

[38] Ramos SA, Massuça LM, Volossovitch A, Ferreira AP, Fragoso I (2021). Morphological and fitness attributes of young male Portuguese basketball players: Normative values according to chronological age and years from peak height velocity. Front. Sports Active Living.

[39] te Wierike SC, de Jong MC, Tromp EJ, Vuijk PJ, Lemmink KA, Malina RM, Elferink-Gemser MT, Visscher C (2014). Development of repeated sprint ability in talented youth basketball players. J. Strength Cond. Res..

[40] Jakovljević, S., Pajić, Z., Gardašević, B. & Višnjić, D. Some anthropometric and power characteristics of 12 and 13 years old soccer and basketball players. *Proceedings 2010* **2**, 42–48. 10.5550/SP.2.2010.06 (2011).

[41] World Medical Association (2013). World Medical Association Declaration of Helsinki: ethical principles for medical research involving human subjects. JAMA.

[42] Moore SA, McKay HA, Macdonald H, Nettlefold L, Baxter-Jones AD, Cameron N, Brasher PM (2015). Enhancing a somatic maturity prediction model. Med. Sci. Sports Exerc..

[43] Marfell-Jones MJ, Stewart AD, De Ridder JH (2012). International Standards for Anthropometric Assessment.

[44] Bompa, T.O., & Haff, G. *Periodization : Theory and Methodology of Training.* 5th edn. (Human Kinetics, 2009).

[45] Verkhoshansky Y, Siff MC (2009). Supertraining.

[46] Kirkendall D, Gruber J, Johnson R (1987). Measurement and Evaluation for Physical Educators.

[47] D’Auria, S., Tanner, R., Sheppard, J. & Manning, J. Evaluation of various methodologies used to assess sprint performance. In *Paper Presented at the Australian Institute of Sport Applied Physiology Conference* (2006).

[48] Earp JE, Newton RU (2012). Advances in electronic timing systems: Considerations for selecting an appropriate timing system. J. Strength Cond. Res..

[49] Stanton, R., Hayman, M., Humphris, N., Borgelt, H., Fox, J., Del Vecchio, L. & Humphries B. Validity of a smartphone-based application for determining sprinting performance. *J. Sports Med. (Hindawi Publ Corp)*. 7476820. 10.1155/2016/7476820 (2016). 10.1155/2016/7476820 PMC4972912 27525305

[50] Buckthorpe M, Morris J, Folland JP (2012). Validity of vertical jump measurement devices. J. Sports Sci..

[51] Brooks ER, Benson AC, Bruce LM (2018). Novel technologies found to be valid and reliable for the measurement of vertical jump height with jump-and-reach testing. J. Strength Cond. Res..

[52] Gabbett T, Kelly J, Pezet T (2007). Relationship between physical fitness and playing ability in rugby league players. J. Strength Cond. Res..

[53] Coutts AJ, Reaburn P, Piva TJ, Rowsell GJ (2007). Monitoring for overreaching in rugby league players. Eur. J. Appl. Physiol..

[54] Bangsbo J, Iaia FM, Krustrup P (2008). The Yo-Yo intermittent recovery test: A useful tool for evaluation of physical performance in intermittent sports. Sports Med..

[55] Fort-Vanmeerhaeghe A, Montalvo A, Latinjak A, Unnithan V (2016). Physical characteristics of elite adolescent female basketball players and their relationship to match performance. J. Hum. Kinet..

[56] Castagna C, Impellizzeri FM, Rampinini E, D'Ottavio S, Manzi V (2008). The Yo-Yo intermittent recovery test in basketball players. J. Sci. Med. Sport..

[57] Koo TK, Li MY (2016). A guideline of selecting and reporting intraclass correlation coefficients for reliability research. J. Chiropr. Med..

[58] Taber KS (2018). The use of Cronbach’s alpha when developing and reporting research instruments in science education. Res. Sci. Educ..

[59] Hopkins, W. G. A scale of magnitudes for effect statistics. Available at: http://www.sportsci.org/resource/stats/index.html (2002). Accessed November 21, 2020.

[60] Goto H, Morris JG, Nevill ME (2019). Influence of biological maturity on the match performance of 8- to 16-year-old, elite, male, youth soccer players. J. Strength Cond. Res..

[61] Cripps AJ, Hopper LS, Joyce C (2016). Coaches’ perceptions of long- term potential are biased by maturational variation. Int. J. Sports Sci. Coach..

[62] Armstrong, N. *Paediatric Exercise Physiology. Advances in Sport and Exercise 187. Science Series*. (Churchill Livingstone Elsevier, 2007).

[63] Gryko K, Kopiczko A, Mikołajec K, Stasny P, Musalek M (2018). Anthropometric variables and somatotype of young and professional male basketball players. Sports (Basel)..

[64] Ramos S, Volossovitch A, Ferreira AP, Fragoso I, Massuça L (2019). Differences in maturity, morphological and physical attributes between players selected to the primary and secondary teams of a Portuguese Basketball elite academy. J. Sports Sci..

[65] Jakovljević S, Karalejić M, Pajić Z, Gardašević B, Mandić R (2011). Influence of anthropometric characteristics on speed abilities of 14 years old elite male basketball players. J. Phys. Educ. Sport..

[66] Rinaldo N, Toselli S, Gualdi-Russo E, Zedda N, Zaccagni L (2020). Effects of anthropometric growth and basketball experience on physical performance in pre-adolescent male players. Int. J. Environ. Res. Public Health.

[67] Nikolaidis PT, Asadi A, Santos EJ, Calleja-González J, Padulo J, Chtourou H, Zemkova E (2015). Relationship of body mass status with running and jumping performances in young basketball players. Muscles Ligaments Tendons J..

[68] Sherar LB, Cumming SP, Malina RM (2010). Adolescent biological maturity and physical activity: Biology meets behavior. Pediatr. Exer. Sci..

[69] Arede J, Esteves P, Ferreira AP, Sampaio J, Leite N (2019). Jump higher, run faster: effects of diversified sport participation on talent identification and selection in youth basketball. J. Sports Sci..

[70] Malina RM, Eisenmann JC, Cumming SP, Ribeiro B, Aroso J (2004). Maturity-associated variation in the growth and functional capacities of youth football (soccer) players aged 13–15 years. Eur. J. Appl. Physiol..

[71] Carvalho, H.M., Coelho-e-Silva, M.J., Eisenmann, J.C., Malina & R.M. Aerobic fitness, maturation, and training experience in youth basketball. *Int. J. Sports Physiol. Perform*. **8(4)**, 428–34. 10.1123/ijspp.8.4.428 (2013). 10.1123/ijspp.8.4.428 23239685

[72] Cumming SP, Lloyd RS, Oliver JL, Eisenmann JC, Malina RM (2017). Bio-banding in sport: Applications to competition, talent identification, and strength and conditioning of youth athletes. Strength Cond. J..

[73] Lloyd RS, Oliver JL (2014). Strenght and Conditioning for Young Athletes: Science and Application.

[74] Schmitz B, Pfeifer C, Kreitz K, Borowski M, Faldum A, Brand SM (2018). The Yo-Yo intermittent tests: A systematic review and structured compendium of test results. Front. Physiol..

[75] Malina RM, Kozieł SM (2014). Validation of maturity offset in a longitudinal sample of Polish boys. J. Sports Sci..

[76] Malina, R.M., Rogol, A.D., Cumming, S.P., Coelho e Silva, M.J. & Figueiredo, A.J. Biological maturation of youth athletes: assessment and implications. *Br. J. Sports Med*. **49(13)**, 852–859. 10.1136/bjsports-2015-094623 (2015). 10.1136/bjsports-2015-094623 26084525

[77] Subijana CL, Lorenzo-Calvo J (2018). Relative age effect and long-term success in the Spanish soccer and basketball national teams. J. Hum. Kinet..

